# Mortality Attributable to Seasonal and Pandemic Influenza, Australia, 2003 to 2009, Using a Novel Time Series Smoothing Approach

**DOI:** 10.1371/journal.pone.0064734

**Published:** 2013-06-03

**Authors:** David J. Muscatello, Anthony T. Newall, Dominic E. Dwyer, C. Raina MacIntyre

**Affiliations:** 1 School of Public Health and Community Medicine, University of New South Wales, Kensington, New South Wales, Australia; 2 Centre for Infectious Diseases and Microbiology Laboratory Services, Institute of Clinical Pathology and Medical Research, Westmead Hospital and Sydney Institute for Emerging Infections and Biosecurity, The University of Sydney, Sydney, New South Wales, Australia; University of Hong Kong, Hong Kong

## Abstract

**Background:**

Official statistics under-estimate influenza deaths. Time series methods allow the estimation of influenza-attributable mortality. The methods often model background, non-influenza mortality using a cyclic, harmonic regression model based on the Serfling approach. This approach assumes that the seasonal pattern of non-influenza mortality is the same each year, which may not always be accurate.

**Aim:**

To estimate Australian seasonal and pandemic influenza-attributable mortality from 2003 to 2009, and to assess a more flexible influenza mortality estimation approach.

**Methods:**

We used a semi-parametric generalized additive model (GAM) to replace the conventional seasonal harmonic terms with a smoothing spline of time (‘spline model’) to estimate influenza-attributable respiratory, respiratory and circulatory, and all-cause mortality in persons aged <65 and ≥65 years. Influenza A(H1N1)pdm09, seasonal influenza A and B virus laboratory detection time series were used as independent variables. Model fit and estimates were compared with those of a harmonic model.

**Results:**

Compared with the harmonic model, the spline model improved model fit by up to 20%. In <65 year-olds, the estimated respiratory mortality attributable to pandemic influenza A(H1N1)pdm09 was 0.5 (95% confidence interval (CI), 0.3, 0.7) per 100,000; similar to that of the years with the highest seasonal influenza A mortality, 2003 and 2007 (A/H3N2 years). In ≥65 year-olds, the highest annual seasonal influenza A mortality estimate was 25.8 (95% CI 22.2, 29.5) per 100,000 in 2003, five-fold higher than the non-statistically significant 2009 pandemic influenza estimate in that age group. Seasonal influenza B mortality estimates were negligible.

**Conclusions:**

The spline model achieved a better model fit. The study provides additional evidence that seasonal influenza, particularly A/H3N2, remains an important cause of mortality in Australia and that the epidemic of pandemic influenza A (H1N1)pdm09 virus in 2009 did not result in mortality greater than seasonal A/H3N2 influenza mortality, even in younger age groups.

## Introduction

Since the work of Farr examining influenza in 1848 [1], it has been recognised that mortality due to influenza will be under-ascertained and misclassified in official mortality statistics [2], [3]. This limitation of official statistics is still evident. For example, between 1997 and 2004 in Australia, official statistics reported an average 83 deaths annually with influenza listed as the underlying cause [4]. This compares with time series estimates of more than 2000 influenza-attributable all-cause deaths per year in persons aged ≥50 years alone [5]. Even during the influenza A(H1N1) pdm09 pandemic in 2009, the global number of deaths from laboratory-confirmed influenza reported to the World Health Organization (WHO) was fifteen times lower than the estimated number of influenza-attributable circulatory and respiratory deaths obtained using modelling [6].

Several reviews and comparisons of time series modelling methods for estimating mortality attributable to influenza have been published in recent years [5], [7]–[9]. At the highest level, methods can be distinguished according to whether they use relatively specific influenza surveillance time series as independent variables to estimate the component of the mortality time series associated with influenza, or whether they assume that influenza is the primary cause of elevated mortality over the predictable seasonal baseline during the cooler months or defined epidemic periods [5], [10]–[12].

The use of an influenza surveillance time series as an independent variable has two major advantages. Firstly, while a statistical association cannot be considered causal, the identification of correlation between the influenza surveillance time series and mortality provides more convincing evidence than the alternative assumption. The alternative assumption is that, during influenza epidemics, influenza is the only factor responsible for any mortality that exceeds some estimated background or baseline level. Secondly, the use of influenza surveillance time series avoids the need to make potentially subjective decisions about criteria used to define the start and end of epidemic periods.

The next important characteristic of the methods is how they control for the annual seasonal winter increase in non-influenza mortality that occurs in temperate weather countries. Non-influenza mortality, which we will call background mortality, is frequently assumed to be cyclical in nature and to follow a sinusoidal wave, that is, a seasonal harmonic pattern [13]. While respiratory and even all-cause mortality time series do show a clear annual winter increase in temperate countries, the inflexibility of the harmonic model assumes that the seasonal pattern of incidence of all non-influenza causes of mortality remains static from year to year. This rigidity could lead to over- or under-estimation of background mortality in any particular year.

Australia lacks an estimate of influenza-attributable mortality for the first year of circulation of influenza A(H1N1)pdm09, as well as recent estimates for seasonal influenza. We produced these estimates for the years 2003 to 2009, in two age groups, under and over 65 years. To achieve this, we used a variation of the harmonic approach in which non-influenza, background mortality seasonality and trend was modelled using a flexible natural cubic spline instead of the more rigid sinusoidal curve. Splines have been used previously in influenza mortality estimation, for removing secular trends in long mortality time series [14]–[16], to assess relative mortality risk associated with influenza [17], to allow non-linear inclusion of confounding variables such as temperature and humidity [15], and to flexibly model short time-scale, background, non-influenza mortality variation [18]. To our knowledge this is the first direct comparison of influenza-associated mortality using both a harmonic modelling and short time-scale spline approach.

## Materials and Methods

We estimated the annual rate and count of influenza-associated deaths using a semi-parametric generalized additive model with influenza virology time series as parametric independent variables and a natural cubic smoothing spline of time as a non-parametric variable (‘spline model’). Generalized additive models (GAM) [19] extend parametric generalized linear models [20] to allow inclusion of non-parametric smoothing functions of one or more independent variables. This is useful when the association between the independent and the dependent variable is non-linear. The additional inclusion of unsmoothed variables as conventional model parameters, such as the influenza surveillance time series in our case, leads to the description of this model type as semi-parametric [19]. These methods have previously been used in environmental epidemiology [21]–[25]. A useful description of methodological issues and alternatives in time series analyses of this kind is given in Schwarz et al [26] and Peng et al [27].

Separate models were fit for each of two age groups, <65 and ≥65 years, and for each of three mortality outcomes as dependent variables; weekly population rates of respiratory, respiratory and circulatory combined, and all-cause deaths. The models included parametric independent variables that comprised time series of weekly counts of influenza virology (laboratory confirmation) surveillance time series indicating the incidence of confirmed infection with influenza A (H1N1)pdm09, seasonal influenza A and, where possible, seasonal influenza B. In this case, a smoothing spline of time (represented by consecutive week number) was used to control for any variance arising from seasonal and time varying non-influenza mortality. Failing to control for this additional variance can lead to model residuals that do not satisfy the important modelling assumption of independence because the residuals are correlated with each other, that is, autocorrelated. In time series analysis, the presence of autocorrelation resulting from time trends and seasonality, can lead to identification of spurious correlations and incorrectly estimated standard errors of model parameter estimates [28]–[30]. Lack of serial independence over time in the residuals was checked using autocorrelation plots.

The spline model equation was:
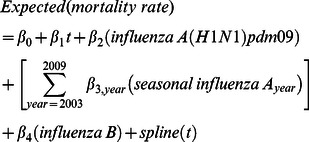
where 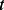 was the sequential week number of the weekly time series observations (not epidemiological week number, which resets every year). The *seasonal influenza A year* variable was the annual seasonal influenza A virology (laboratory confirmation) variable split into years, such that for each year, the virology count was set to zero in all other years (Table S1 in File S1).

**Figure 1:**
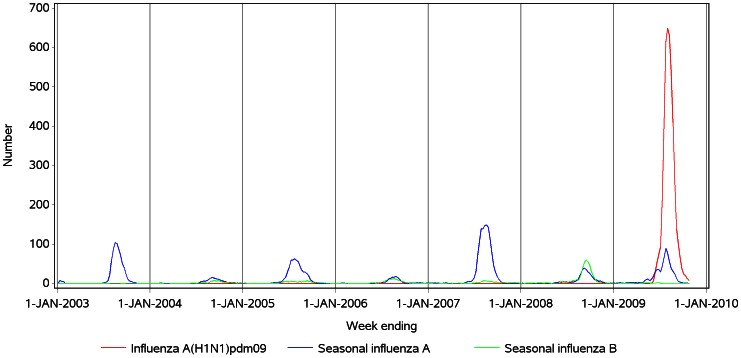
Influenza laboratory detection time series, persons of all ages, Australia, 2003 to 2009. Notes: 1. The values shown are the moving average of the current and previous two weeks, as used in the regression models (see Table S1 in File S1).

The flexibility of the spline curve is determined by the degrees of freedom specified in the model. We specified 43 degrees of freedom which achieved control of autocorrelation, a criterion often used in studies that employ these models [27]. One degree of freedom is allocated to the parametric term for linear time (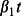) [31] and approximately 6 per year for the spline. This accounted for time variation over approximately two-month intervals, and provided control of autocorrelation in the model residuals, while leaving variation at less than two months available for correlating with the independent virology variables. Since influenza epidemics in our country typically span only a few months (Figure 1), this degree of control for trend and seasonality still allowed for adequate assessment of the association between influenza and mortality.

To avoid estimation problems previously reported with default options provided in standard GAM software, we included the following model options in the SAS GAM procedure: EPSILON = 1E-15, EPSSCORE = 1E-15, MAXITER = 1000, and MAXITSCORE = 1000 [25].

To assess the model, we compared it with a more conventional harmonic model, which controls for seasonally varying non-influenza mortality using pre-calculated sine and cosine (harmonic) terms as parametric independent variables [13]:
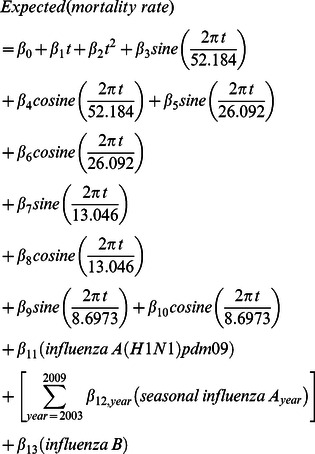
where 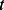 was the sequential week number as above. The terms 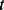 and 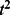 were included to accommodate long-term linear and curvilinear secular trends, respectively. The denominator of each sine and cosine term represents the periodicity of the seasonal wave pattern in the same units as 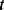. In this case we used 52.184, 26.092, 13.046, and 8.6973 weeks to represent four sine waves for predictable annual, semi-annual, quarterly and bi-monthly variability, respectively. These periods were calculated across the entire study period, taking into account leap years.

To assess the fit of each model, the square root of the mean squared error (RMSE) was used, which summarises the overall differences from the modelled values and the observed values (‘model residuals’) over the entire time series in the same units as the observed outcome values. A lower value means a better fit.

**Figure 2:**
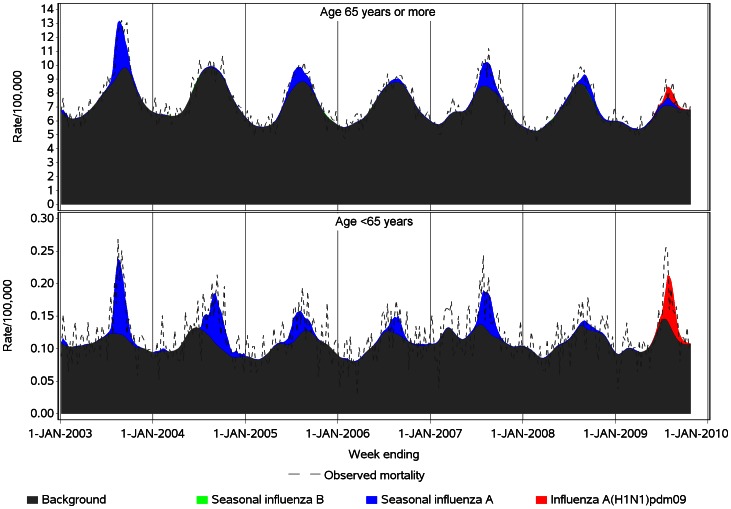
Spline model estimates of weekly influenza and non-influenza-attributable respiratory mortality in persons aged <65 and ≥65, by virus type, Australia, 2003 to 2009. Notes: 1. The influenza A laboratory detection time series was included in the regression models as a separate variable for each year, to allow for variation in laboratory reporting and testing methods, virulence of influenza strains and susceptibility of the population (see Table S1 in File S1). 2. For 2009, the seasonal influenza A laboratory detection time series was unable to be included in the regression model for <65 year-olds (see Table S1 in File S1).

**Figure 3:**
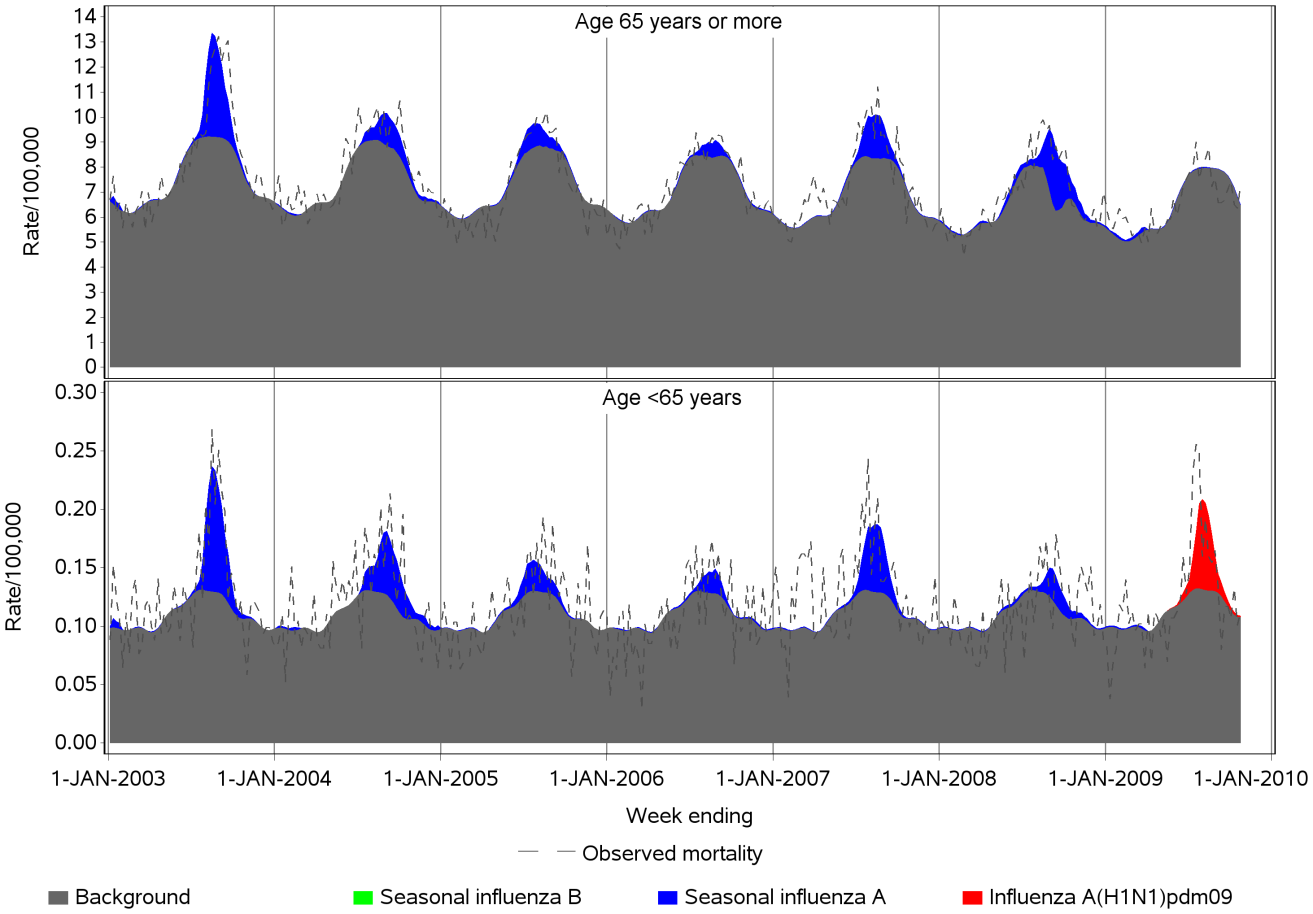
Harmonic model estimates of weekly influenza and non-influenza-attributable respiratory mortality in persons aged <65 and ≥65, by virus type, Australia, 2003 to 2009. Notes: 1. The influenza A laboratory detection time series was included in the regression models as a separate variable for each year, to allow for variation in laboratory reporting and testing methods, virulence of influenza strains and susceptibility of the population (see Table S1 in File S1). 2. For 2009, the seasonal influenza A laboratory detection time series was unable to be included in the regression model for <65 year-olds (see Table S1 in File S1).

The estimated weekly component of predicted mortality due to non-influenza background mortality, as shown in Figures 2 and 3, was obtained from each model by using the model equation to calculate the predicted mortality when all influenza virology variables were set to zero. The estimated weekly component of mortality associated with each influenza variable was obtained by multiplying the variable’s parameter estimate by the observed value of that variable in each week. The weekly estimated rates were summed over the year to obtain annual rates.

Methods for calculation of confidence intervals and all-age mortality estimates are described in File S1. All models were fit using the GAM procedure in SAS version 9.3. The harmonic model used the fully parametric capability of the procedure. In all models, normally distributed model residuals was assumed and this was checked using quantile-quantile plots of the residuals.

### Data Sources and Preparation

For the calendar years 2003 through 2009, time series of weekly counts of Australian deaths were obtained from the Australian Bureau of Statistics. Time series for deaths with an underlying cause certified as respiratory disease (Chapter X, J codes, of the International Classification of Diseases, Revision 10), respiratory and circulatory deaths combined (Chapters IX and X, I and J codes), and total (all-cause) deaths. Age-specific mortality rates per 100,000 population were calculated using denominators of weekly population estimates linearly interpolated from published annual mid-year resident population estimates [32].

For the same period, time series were prepared of weekly counts of influenza infections detected in humans by laboratories designated as National Influenza Centres in Australia by the WHO and reported to its FluNet surveillance program. Weekly counts were collated by week of specimen collection from the patient. Values were downloaded from the FluNet public web site [33]. A time series was prepared for each of seasonal influenza A, seasonal influenza B and influenza A(H1N1)pdm09. There were five weeks at the end of 2005 (summer in Australia) that were missing from the FluNet data. Counts for these weeks were replaced with zeros. The proportion of influenza A in the FluNet data that was unsubtyped ranged from 10% in 2008 to 85% in 2006, thus preventing separation of seasonal influenza A into H1 and H3 subtypes.

Table S1 in File S1 summarises the methodological decisions made in response to various data issues and analysis complexities.

### Ethics

This study used publicly available virology (laboratory confirmation) counts and aggregated mortality counts provided by the national vital statistics bureau, therefore ethics approval was not required.

## Results

### Model Comparisons (Spline and Harmonic)

Figure 1 shows the moving average influenza virus detection counts by week over the study period. High counts of pandemic influenza detection occurred during 2009. Figures 2 and 3 show the spline and harmonic model results, respectively, for the respiratory mortality outcome. The greater flexibility of the spline is particularly evident in the <65 year-old group, in which the seasonal pattern of non-influenza mortality is less distinct. Compared with the harmonic regression model, the spline model with smoothing spline improved model fit, as measured by the root mean square error statistic (RMSE), by between 7% and 10% in <65 year-olds, and between 17% and 20% in persons aged ≥65 years (Table 1). In the over 65 years age group, unlike the spline model, the residuals of the harmonic model retained some non-independence, with statistically significant first order autocorrelation coefficients of >0.3 for all three outcome variables.

**Table 1:** Root mean square error (RMSE) of the overall mortality rate/100,000 predicted by each model, for the spline and harmonic models.

		Model		
Mortality outcome	Age group (years)	Spline	Harmonic	Difference - spline versus harmonic (%)
Respiratory	<65	0.46	0.50	−9.3
	≥65	9.72	11.69	−16.8
Respiratory+circulatory*	<65	1.17	1.26	−6.7
	≥65	23.75	29.77	−20.2
All cause*	<65	2.48	2.77	−10.4
	≥65	35.75	44.47	−19.6

* Influenza B was excluded from these models (see Table S1 in File S1).

Estimates of influenza-attributable mortality between the spline and harmonic model for each of three mortality outcomes were compared (rates are presented in Tables 2–4 and numbers in Tables S2–S4 in File S1).

**Table 2:** Estimated influenza-attributable respiratory mortality rate, by influenza virus, model type and age, Australia, 2003 to 2009.

		Rate/100,000 population (95% confidence interval)					
		Influenza A(H1N1)pdm09		Seasonal influenza A		Seasonal influenza B	
		Model		Model		Model	
Age group (years)	Year	Spline	Harmonic	Spline	Harmonic	Spline	Harmonic
<65	**2003**	n/a	n/a	0.8 (0.6, 1.0)	0.7 (0.5, 0.9)	0.0 (0.0, 0.0)	0.0 (0.0, 0.0)
	**2004**	n/a	n/a	0.8 (0.5, 1.0)	0.5 (0.3, 0.8)	0.0 (−0.1, 0.1)	0.0 (−0.1, 0.1)
	**2005**	n/a	n/a	0.4 (0.2, 0.6)	0.3 (0.0, 0.5)	0.0 (−0.1, 0.1)	0.0 (−0.2, 0.1)
	**2006**	n/a	n/a	0.2 (0.0, 0.4)	0.2 (−0.1, 0.4)	0.0 (−0.2, 0.1)	0.0 (−0.2, 0.2)
	**2007**	n/a	n/a	0.5 (0.3, 0.7)	0.5 (0.3, 0.7)	0.0 (−0.1, 0.1)	0.0 (−0.1, 0.1)
	**2008**	n/a	n/a	0.1 (−0.6, 0.8)	0.2 (−0.5, 1.0)	−0.1 (−0.7, 0.5)	0.0 (−0.7, 0.6)
	**2009**	0.5 (0.3, 0.7)	0.5 (0.3, 0.7)	0.0 (0.0, 0.0)	0.0 (0.0, 0.1)	0.0 (0.0, 0.0)	0.0 (0.0, 0.0)
≥65	**2003**	n/a	n/a	25.8 (22.2, 29.5)	28.4 (23.5, 33.4)	0.0 (−0.1, 0.1)	−0.1 (−0.3, 0.0)
	**2004**	n/a	n/a	0.7 (−4.2, 5.7)	12.9 (6.5, 19.3)	0.1 (−2.1, 2.2)	−2.4 (−5.1, 0.3)
	**2005**	n/a	n/a	11.7 (7.0, 16.3)	9.3 (3.2, 15.4)	0.1 (−2.8, 3.0)	−3.2 (−6.8, 0.4)
	**2006**	n/a	n/a	2.0 (−3.0, 7.0)	5.1 (−1.4, 11.5)	0.1 (−3.3, 3.5)	−3.8 (−8.0, 0.4)
	**2007**	n/a	n/a	14.9 (10.8, 19.0)	14.8 (9.4, 20.3)	0.1 (−2.4, 2.5)	−2.7 (−5.7, 0.3)
	**2008**	n/a	n/a	7.8 (−6.5, 22.1)	23.3 (5.8, 40.7)	0.4 (−12.2, 13.0)	−13.9 (−29.5, 1.6)
	**2009**	5.2 (−2.8, 13.2)	−0.2 (−10.1, 9.7)	4.8 (−5.8, 15.3)	1.5 (−11.4, 14.5)	0.0 (−0.4, 0.5)	−0.5 (−1.1, 0.1)

**Table 3:** Estimated influenza-attributable respiratory and circulatory mortality rate, by influenza virus, model type and age, Australia, 2003 to 2009.

		Rate/100,000 population (95% confidence interval)			
		Influenza A(H1N1)pdm09		Seasonal influenza A	
		Model		Model	
Age group (years)	Year	Spline	Harmonic	Spline	Harmonic
<65	**2003**	n/a	n/a	1.0 (0.6, 1.5)	1.3 (0.8, 1.9)
	**2004**	n/a	n/a	1.2 (0.6, 1.7)	0.7 (0.0, 1.4)
	**2005**	n/a	n/a	0.5 (−0.1, 1.0)	0.3 (−0.3, 1.0)
	**2006**	n/a	n/a	0.1 (−0.4, 0.6)	−0.2 (−0.8, 0.4)
	**2007**	n/a	n/a	1.0 (0.5, 1.4)	0.9 (0.4, 1.5)
	**2008**	n/a	n/a	−0.5 (−1.0, 0.1)	0.2 (−0.4, 0.8)
	**2009**	0.6 (0.2, 1.1)	0.2 (−0.4, 0.7)	0.0 (−0.1, 0.0)	0.0 (0.0, 0.0)
≥65	**2003**	n/a	n/a	53.9 (45.0, 62.8)	67.3 (54.7, 79.8)
	**2004**	n/a	n/a	17.2 (6.0, 28.5)	33.9 (18.0, 49.7)
	**2005**	n/a	n/a	46.0 (35.6, 56.4)	21.7 (6.9, 36.6)
	**2006**	n/a	n/a	9.7 (0.0, 19.4)	−4.1 (−18.1, 10.0)
	**2007**	n/a	n/a	35.3 (26.0, 44.6)	38.4 (25.1, 51.6)
	**2008**	n/a	n/a	32.4 (21.2, 43.5)	39.3 (24.4, 54.3)
	**2009**	7.9 (−11.6, 27.4)	−2.6 (−27.7, 22.5)	31.4 (7.1, 55.8)	−9.1 (−40.2, 22.0)

Note: Influenza B was excluded from the analysis for this outcome (see Table S1 in File S1).

**Table 4:** Estimated influenza-attributable all-cause mortality rate, by influenza virus, model type and age, Australia, 2003 to 2009.

		Rate/100,000 population (95% confidence interval)			
		Influenza A(H1N1)pdm09		Seasonal influenza A	
		Model		Model	
Age group (years)	Year	Spline	Harmonic	Spline	Harmonic
<65	**2003**	n/a	n/a	2.1 (1.2, 3.0)	2.3 (1.1, 3.5)
	**2004**	n/a	n/a	1.9 (0.8, 3.1)	0.8 (−0.6, 2.3)
	**2005**	n/a	n/a	−0.7 (−1.8, 0.4)	−0.4 (−1.8, 0.9)
	**2006**	n/a	n/a	−1.1 (−2.1, −0.1)	−0.5 (−1.8, 0.8)
	**2007**	n/a	n/a	1.7 (0.8, 2.7)	2.0 (0.7, 3.2)
	**2008**	n/a	n/a	0.7 (−0.5, 1.8)	1.1 (−0.3, 2.5)
	**2009**	0.2 (−0.8, 1.1)	0.1 (−1.0, 1.3)	0.0 (0.0, 0.1)	0.1 (0.0, 0.1)
≥65	**2003**	n/a	n/a	79.0 (65.5, 92.4)	88.2 (69.5, 107.0)
	**2004**	n/a	n/a	40.9 (24.0, 57.8)	41.8 (18.1, 65.5)
	**2005**	n/a	n/a	57.1 (41.5, 72.8)	7.2 (−14.9, 29.4)
	**2006**	n/a	n/a	9.3 (−5.3, 23.9)	−14.4 (−35.4, 6.6)
	**2007**	n/a	n/a	49.8 (35.8, 63.8)	49.7 (29.9, 69.4)
	**2008**	n/a	n/a	40.9 (24.1, 57.7)	71.0 (48.7, 93.3)
	**2009**	17.6 (−11.8, 47.0)	−9.6 (−47.0, 27.9)	31.6 (−5.0, 68.2)	−16.2 (−62.6, 30.2)

Note: Influenza B was excluded from the analysis for this outcome (see Table S1 in File S1).

Unlike the spline model, the harmonic model produced negative estimates of influenza A(H1N1)pdm09-attributable mortality for all outcomes in persons aged ≥65 years. Even so, the positive estimates from the spline model in that age group were not statistically significant (lower 95% confidence limit ≤0) (Tables 2–4, and Tables S2–S4 in File S1). Influenza B respiratory mortality estimates from the harmonic model in ≥65 year-olds were negative in all years. In <65 year-olds, both models produced small negative estimates of influenza B respiratory mortality (Tables 2 and Table S2 in File S1).

Given its improved fit and more stable results, only results from the spline model are reported below in terms of the estimates of influenza-attributable mortality in Australia.

### Estimates of Influenza-attributable Mortality

For pandemic influenza in 2009, the statistically significant estimates for <65 year-olds produced by the spline model were 0.5 (95% confidence interval (CI) 0.3, 0.7) and 0.6 (95% CI 0.2, 1.1) per 100,000 for respiratory only, and respiratory and circulatory mortality, respectively. For all-cause mortality, the non-statistically significant pandemic estimate, 0.2 (95% CI −0.8, 1.1) was smaller than the equivalent estimate from the respiratory model, but its confidence interval did overlap the confidence intervals of the estimates for the more specific outcomes. For ≥65 year-olds, the model did not produce statistically significant pandemic influenza estimates for any outcome, although the estimates of 5.2 (95% CI −2.8, 13.2), 7.9 (95% CI −11.6, 27.4) and 17.6 (95% CI −11.8, 47.0) per 100,000 for respiratory only, respiratory and circulatory, and all-cause mortality, respectively, showed an increase as the outcome broadened, as did the all-age estimates of 1.1 (95% CI 0.0, 2.2), 1.6 (95% CI −1.1, 4.3) and 2.5 (95% CI −1.5, 6.5) per 100,000, respectively (Tables 2–4, and Tables S2–S4 in File S1). The number of all-cause pandemic deaths estimated for Australia in persons of all ages was 543 (95% CI −334, 1421), although this was not statistically significant (Table 5).

**Table 5:** Estimated all-age influenza-attributable mortality rate from the spline model, by mortality outcome and influenza virus, Australia, 2003 to 2009.

		Influenza A(H1N1)pdm09		Seasonal influenza A		Seasonal influenza B*	
Mortality outcome	Year	Rate/100,000	Count	Rate/100,000	Count	Rate/100,000	Count
Respiratory	**2003**	n/a	n/a	4.0 (3.5, 4.5)	792 (695, 889)	0.0 (0.0, 0.0)	0 (−3, 3)
	**2004**	n/a	n/a	0.8 (0.1, 1.4)	152 (18, 286)	0.0 (−0.3, 0.3)	2 (−54, 58)
	**2005**	n/a	n/a	1.9 (1.2, 2.5)	375 (247, 503)	0.0 (−0.4, 0.4)	3 (−74, 79)
	**2006**	n/a	n/a	0.4 (−0.2, 1.1)	92 (−49, 233)	0.0 (−0.4, 0.5)	3 (−89, 95)
	**2007**	n/a	n/a	2.4 (1.8, 3.0)	503 (384, 622)	0.0 (−0.3, 0.3)	2 (−65, 70)
	**2008**	n/a	n/a	1.1 (−0.9, 3.1)	240 (−185, 665)	0.1 (−1.6, 1.7)	12 (−346, 370)
	**2009**	1.1 (0.0, 2.2)	247 (10, 483)	0.6 (−0.8, 2.1)	140 (−169, 449)	0.0 (−0.1, 0.1)	0 (−13, 14)
Respiratory and circulatory	**2003**	n/a	n/a	7.9 (6.7, 9.1)	1,548 (1,309, 1,787)	n/a	n/a
	**2004**	n/a	n/a	3.2 (1.7, 4.8)	647 (340, 953)	n/a	n/a
	**2005**	n/a	n/a	6.4 (5.0, 7.9)	1,295 (1,007, 1,584)	n/a	n/a
	**2006**	n/a	n/a	1.4 (0.0, 2.7)	278 (2, 554)	n/a	n/a
	**2007**	n/a	n/a	5.6 (4.3, 6.9)	1,157 (886, 1,428)	n/a	n/a
	**2008**	n/a	n/a	4.3 (2.8, 5.8)	921 (603, 1,238)	n/a	n/a
	**2009**	1.6 (−1.1, 4.3)	349 (−228, 926)	4.2 (1.0, 7.5)	917 (208, 1,627)	n/a	n/a
All-cause	**2003**	n/a	n/a	12.0 (10.1, 13.9)	2,365 (1,988, 2,741)	n/a	n/a
	**2004**	n/a	n/a	7.0 (4.6, 9.4)	1,394 (911, 1,877)	n/a	n/a
	**2005**	n/a	n/a	7.5 (5.4, 9.5)	1,509 (1,096, 1,922)	n/a	n/a
	**2006**	n/a	n/a	1.2 (−0.7, 3.2)	251 (−145, 646)	n/a	n/a
	**2007**	n/a	n/a	8.1 (6.1, 10.2)	1,692 (1,266, 2,118)	n/a	n/a
	**2008**	n/a	n/a	6.0 (3.6, 8.5)	1,286 (762, 1,810)	n/a	n/a
	**2009**	2.5 (−1.5, 6.5)	543 (−334, 1,421)	4.3 (−0.7, 9.2)	928 (−143, 1,998)	n/a	n/a

* Influenza B was excluded from the respiratory and circulatory, and all-cause mortality analyses (see Table S1 in File S1).

In <65 year-olds, the estimates for seasonal influenza A respiratory only, and respiratory and circulatory, mortality in 2003 and 2007, the two largest seasonal influenza A years, were not statistically significantly different from the estimated pandemic-attributable mortality in 2009 for those outcomes. In persons aged ≥65 years, estimated seasonal influenza A-attributable respiratory, respiratory and circulatory, and all-cause mortality in 2003 significantly exceeded the 2009 pandemic influenza mortality estimates in that age group. This was also found for the equivalent all-age estimates (Tables 2, 3, and Tables S2, S3 in File S1).

Table 5 shows the all-age estimates obtained by combining the <65 and ≥65 estimates for each mortality outcome, only for the spline model. The maximum number of deaths in persons of all ages estimated for seasonal influenza A was 2,365 (95% CI 1,988, 2,741) in 2003. This represented a population mortality rate of 12.0 (95% CI 10.1, 13.9) per 100,000. In that year, all-age respiratory, respiratory and circulatory, and all-cause mortality were statistically significantly greater than estimated pandemic mortality in 2009. Only 2003 had an estimated all-age seasonal influenza A-attributable mortality estimate that was statistically significantly greater than the estimate for the 2009 pandemic strain for all three mortality outcomes examined. The 2003 influenza-attributable mortality estimates represented 6.7% of all respiratory mortality, 2.6% of respiratory and circulatory mortality and 1.8% of all-cause mortality in that year. In the same year, <65 year-olds accounted for 17%, 12% and 15% of the estimated respiratory, respiratory and circulatory and all-cause mortality, respectively.

The average annual proportion of all-age, all-cause mortality attributable to influenza for the seasonal influenza years 2003–2008 was 1.1%. For all-age respiratory, and respiratory and circulatory mortality, the corresponding proportions were 3.2% and 1.7%, respectively.

Estimated influenza B-attributable respiratory mortality was negligible in all years (Table 2), and including it in models for the respiratory and circulatory, and all-cause outcomes did not allow a suitable model to be fit and was thus excluded from those models (see Table S1 in File S1).

## Discussion

The spline model produced a better model fit and, unlike the harmonic model, produced non-negative estimates of influenza A(H1N1)pdm09-attributable mortality for all outcomes and of influenza B-attributable respiratory mortality in ≥65 year-olds. For ≥65 year-olds, the spline model estimates of 2009 pandemic influenza mortality increased consistently with the broadening of the mortality outcome measure, although these were not statistically significant. Neither model was able to produce statistically significant estimates for every scenario evaluated. This may reflect a combination of Australia’s relatively small population of approximately 22 million in 2009, and relatively low risk of death at any age and long life expectancies [34]. These factors present a challenge for estimation of influenza-attributable mortality through time series analysis and require models that produce the best possible fit.

The best estimate of all-cause, all-age mortality attributable to the epidemic of influenza A(H1N1)pdm09 in 2009 provided by this study was approximately 550 deaths, or a rate of 2.5 per 100,000 population, although this estimate was not statistically significant. For respiratory and circulatory mortality, the best, but also non-statistically significant estimate was approximately 350 deaths (4.2 per 100,000). The respiratory mortality outcome provided a statistically significant all-age estimate of 247 deaths (1.1 per 100,000), of which 95 (38% of all-age deaths, 0.5 per 100,000) were in persons aged <65 years. Given that the role of pandemic influenza infection in deaths was probably incompletely ascertained in 2009 and was more likely to be identified in patients presenting with primarily respiratory illness, the estimate compares plausibly with the recorded number of laboratory-confirmed pandemic deaths of 190 [35], and is within the range estimated in a global mortality modelling study [6]. The lower proportion of deaths in the <65 age group (38%) contrasts with the 71% reported in laboratory confirmed pandemic deaths in the most populous state of Australia for this age group [36]. This discrepancy could reflect laboratory testing bias towards younger persons.

Unlike the respiratory mortality estimate, our respiratory and circulatory all-cause pandemic mortality estimate for 2009 was not statistically significant. This may have meant that unlike seasonal influenza, the effect of pandemic influenza on mortality was more directly observed through respiratory illness and was thus diluted when observed through all-cause mortality. This observation is supported by estimates from Mexico [37], but not from France [38]. Relatively high rates of laboratory testing in 2009 among people with respiratory illness may have led to a more evident correlation between our laboratory and respiratory mortality time series. This may also reflect the younger age profile of infection with the pandemic strain, as co-morbidities are less common among younger persons [39]. In addition, younger people are more likely to have received intensive care if critically ill with pandemic infuenza, than elderly persons who are more likely to suffer severe outcomes during severe seasonal influenza epidemics. In Australia, use of critical care for respiratory illness in younger adults was clearly greater during the epidemic of the pandemic influenza virus in 2009 than during prior seasonal epidemics [40]. Availability of critical care technologies such as extracorporeal membrane oxygenation (ECMO) is also thought to have had an impact on survival [41].

The 2003 winter was clearly the most severe season of the years studied, with an estimate of approximately 2400 (12 per 100,000) influenza A-attributable all-cause deaths. Of these, 15% were in persons aged <65 years. The 2003 season was characterised by an epidemic of the influenza A/Fujian/411/2002 (H3N2)-like virus strain which caused widespread outbreaks internationally and its impact may have been enhanced by a poorly matching H3N2 vaccine strain [42]–[44].

In all non-pandemic years except 2008, influenza A (H3N2) strains predominated (Table 6), so it is impossible from our data to draw a conclusion about severity of H3N2 strains compared with H1N1 strains. In 2003, the predominant influenza strain among specimens available for molecular sub-typing in Australia was A/Fujian/411/2002 (H3N2)-like (WHO Collaborating Centre for Reference and Research on Influenza, Melbourne, Victoria, Australia, personal communication).

**Table 6:** Most frequently identified influenza virus strains among specimens that could be typed, by year, Australia, 2003–2009.

Year	Influenza virus strains (% of typeable specimens)
2003	A/Fujian/411/2002 (H3N2)-like (97%)
2004	A/Fujian/411/2002 (H3N2)-like (50%), A/Wellington/1/2004 (H3N2)-like (24%), B/Shanghai/361/2002 (21%)
2005	A/California/7/2004 (H3N2)-like (37%), A/Wellington/1/2004 (H3N2)-like (22%), A/New Caledonia/20/99 (H1N1)-like (19%), B/Shanghai/361/2002 (12%)
2006	A/Wisconsin/67/2005 (H3N2)-like (59%), B/Malaysia/2506/2004-like (36%)
2007	A/Brisbane/10/2007 (H3N2)-like (40%), A/Solomon Islands/3/2006 (H1N1)-like (32%), A/Wisconsin/67/2005 (H3N2)-like (19%)
2008	B/Florida/4/2006-like (33%), B/Malaysia/2506/2004-like (32%), A/Brisbane/10/2007 (H3N2)-like (22%), A/Brisbane/59/2007 (H1N1)-like (12%)
2009	A/California/7/2009 (H1N1)pdm09-like (70%), A/Brisbane/10/2007 (H3N2)-like (18%)

Source: WHO Collaborating Centre for Reference and Research on Influenza, Melbourne, Australia.

Notes:

1. Specimens supplied to the Collaborating Centre may not be representative of the overall distribution of influenza infections in the population.

2. The proportion of each strain does not reflect the strength of influenza activity in the population.

Several countries have published national pandemic and seasonal influenza mortality comparisons using comparable methods, but the age groups and outcomes examined vary. Point estimates of pandemic influenza-attributable respiratory mortality rates for 2009 in France (0.3/100,000 in <65 years, 4.1/100,000 in ≥65 years, and 1.0/100,000 in all ages) were remarkably similar to ours for Australia [38]. Our point estimates for respiratory and circulatory mortality rates for the pandemic strain were similar to those of Hong Kong, although the mortality rate in ≥65 year-olds was somewhat higher in Hong Kong [15]. For pandemic influenza-attributable all-cause mortality rates, our Australian estimate was above that of England and Wales [45], and Hong Kong [15], similar to that of Denmark [16], and lower than that of The Netherlands [46]. Pandemic estimates for all three mortality outcomes for Mexico were more than four-fold higher than those of other countries including our own [37]. Seasonal influenza estimates in Australia in the years prior to 2009 from our model were generally lower than those from the same studies [15], [16], [37], [38], [45], [46].

The main limitations of our study relate to the influenza laboratory time series data and could explain the difficulty in obtaining meaningful values of influenza-attributable mortality in all groups and years. The mortality data is complete for Australia, but infection trends need to be represented by available surveillance information. The probability of a human influenza infection leading to a laboratory test is small, particularly since the majority of people with an infection do not seek medical care, and only a proportion of those seeking medical attention will be tested. Influenza laboratory testing policies and clinician propensity to test might vary between jurisdictions within Australia. Testing propensity increased dramatically during the A(H1N1)pdm09 epidemic of 2009, particularly in the early stages when the aim was to detect its presence in Australia and to control its spread. The virology information in the FluNet database is reported voluntarily by designated WHO National Influenza Centres, so completeness could vary over time. Only tests that use direct virus detection techniques such as polymerase chain reaction (PCR) are reported. The laboratory tests themselves have varying sensitivity, and the type or quality of specimen collection and delays in collection can lead to false negative results [47]–[49]. Despite these limitations, the FluNet system should give broadly representative influenza epidemic time trends for Australia and has the advantage that the best available virological detection techniques were used and the results can be split by influenza types (A, B and influenza A(H1N1)pdm09). Because of incomplete subtyping, we could not split seasonal influenza A into AH3 and AH1.

Confounders which could have altered the associations we observed include temperature, humidity and other respiratory infections such as RSV. Influenza circulates mainly in winter in Australia, so extreme cold could have explained some excess mortality and led to over-estimation of the influenza effect. On the other hand, the majority of Australia’s population lives in warm temperate regions where extreme cold is uncommon, and the effect of cold temperature on mortality is estimated to be relatively small [16], [50]. Temperature and humidity also appear to play a role in predicting influenza mortality, but the mechanism is unclear; the variables may operate independently of influenza infection, enhance influenza’s communicability or influence the host’s susceptibility to infection [15], [51]. It is likely that the incidence of influenza as measured by laboratory detection of influenza will reflect the final result of any interactions between weather and influenza incidence and so this may not be an important consideration in our study. Finally, we did not have incidence data for other respiratory infections such as respiratory syncytial virus (RSV) which has also been shown to be associated with mortality, but to a lesser degree than influenza [10]. Further, Australia does not have nationally consistent influenza-like illness surveillance, which could enhance estimates of influenza incidence [18]. Studies of this kind are ecological and therefore a causal relationship between influenza circulation and the excess deaths estimated by this study cannot be confirmed.

### Conclusions

When influenza virology (laboratory confirmation) time series are available as independent variables, generalised additive models with a smoothing spline of time offer a method for estimating influenza-attributable mortality that does not require the assumption of perfectly predictable seasonal background mortality trends. Harmonic models assume that the seasonal behaviour of all non-influenza causes of mortality do not vary from year to year. However, a suitably flexible smoothing function effectively accommodates any variance in the observed outcome that remains after the contribution of the influenza terms in the model has been estimated. The lack of gold standard influenza mortality statistics means that the best estimates of influenza-attributable mortality depend on obtaining the best available model.

This analysis provides evidence that pandemic influenza mortality in Australia in 2009 was lower than seasonal influenza mortality in recent years, particularly in ≥65 year-olds. Australian pandemic mortality rates in 2009 were low and broadly similar to those of other advanced economies that have published comparable national studies, but were substantially lower than estimated rates for Mexico. The shift in age-specific mortality towards younger persons characteristic of pandemic influenza was evident, although this did not mean greater mortality in younger persons compared with seasonal influenza A in other years. Prevention of seasonal influenza and consequent mortality, particularly influenza A/H3N2, remains a high priority.

## Supporting Information

File S1 **Methods for calculation of confidence intervals and all-age mortality estimates and Tables S1–S4.**
(PDF)
