# Supplementary material for: Mortality Attributable to Seasonal and Pandemic Influenza, Australia, 2003 to 2009, Using a Novel Time Series Smoothing Approach
Source: PLoS One. 2013 Jun 3;8(6):e64734. doi: 10.1371/journal.pone.0064734 (PMC3670851; doi:10.1371/journal.pone.0064734)
Supplement: File S1 — Methods for calculation of confidence intervals and all-age mortality estimates and Tables S1–S4. (PDF) [file pone.0064734.s001.pdf]

Supplementary File S1

Calculation of confidence intervals and all-age estimates for influenza attributable mortality.

Ninety five per cent confidence intervals of the estimated annual mortality rates attributable to each influenza virus were obtained by repeating the annual influenza-attributable mortality calculations using each of the upper and lower 95% confidence limits of the parameter estimates,

$$\beta \pm (1.96 \times se)$$

where se was the standard error of the parameter estimate, β.

All-age parameter estimates and standard errors for each virus variable were used in the same way as the age-specific estimates to obtain all-age virus-attributable mortality estimates. To obtain the all-age parameter estimate for a virus, $\beta_{all\ age}$, the age-specific parameter estimates for the virus variable, $\beta_{<65}$ and $\beta_{\geq 65}$, were proportionally summed according to the relative contribution of the age group to the total population:

$$\beta_{all\ age} = \frac{(\beta_{<65} \times p_{<65}) + (\beta_{\geq 65} \times p_{\geq 65})}{p_{all\ age}}$$

where $p_{<65}$, $p_{\geq 65}$, and $p_{all\ age}$ were the age-specific and all-age populations respectively.

Confidence intervals for the all-age mortality estimates were calculated in the same way as the age-specific intervals, with the standard error, $se_{all\ age}$, of the all-age parameter estimate obtained as:

$$se_{all\ age} = \sqrt{\left(\frac{se_{<65} \times p_{<65}}{p_{all\ age}}\right)^2 + \left(\frac{se_{\geq 65} \times p_{\geq 65}}{p_{all\ age}}\right)^2}$$

Because it is implausible to have negative mortality associated with influenza when observed virology counts indicated influenza was circulating, negative parameter estimates were excluded from these calculations.

Table S1. Adjustments made to data to facilitate a valid analysis

Issue	Response	Rationale	Comment
Mortality data late in 2009 was incomplete because death registration may not occur in the year of death.	The final two months of 2009 were excluded.	Including incomplete data will alter the shape of the time series and lead to poor model fit.	
Annual variation in completeness of laboratory reporting, influenza strain virulence, influenza vaccine coverage and effectiveness, and variation in propensity to test, especially during the pandemic from 1 April 2009.	Split seasonal influenza A variable into a separate variable for each year that has a value of zero in all other years. The 2008 variable was extended to 31 March 2009, prior to the start of the pandemic.	Allows seasonal influenza A in each year to have a different association with mortality. The early part of 2009 included with the 2008 variable was the warm season so little influenza occurred then anyway.	Not done for influenza B because counts were low in all years. Not done for influenza A(H1N1)pdm09 because it only occurred in 2009. The disadvantage is that it reduces the power to find an association because of the reduced sample size resulting from splitting the seasonal influenza A variable into separate variables.
In 2009, both the spline and harmonic models attribute all or almost all influenza-attributable mortality in under-65 year-olds to seasonal influenza A in 2009. Collinearity was evident between seasonal and pandemic influenza A (r=0.83).	For models in under-65 year-olds, set seasonal influenza A to zero from 1 April 2009 when pandemic started.	Epidemiology of influenza A(H1N1)pdm09 infections in 2009 showed that circulation was far greater in under-65 year-olds. Seasonal influenza A is mostly associated with mortality in the elderly.	For respiratory deaths, the spline and harmonic models were able to discriminate between seasonal and pandemic influenza in 2009, but the estimated pandemic mortality was negligible. For the other mortality outcomes, all mortality was attributed to seasonal influenza A in under-65 year-olds. This might over-estimate the pandemic impact, because there was some seasonal A impact in this age group in many years.
There may be a lag between the incidence of infection as measured by patient sample testing and the incidence of death due to influenza infection.	Use a moving average of the current and previous 2 weeks' laboratory results for all virology time series variables.	Intervals from symptom onset to death from pandemic influenza have been estimated at 12 days [1,2] and from hospitalisation to death in seasonal influenza at 7 days [3].	

Issue	Response	Rationale	Comment
Epidemiological weeks 2005: 47-52 were missing from the Australian FluNet database.	Replace missing virology counts values with zeros.	The missing weeks were in summer, when influenza incidence is likely to be very low.	
Population size and distribution varies over time.	Calculate population rates each week for the mortality outcome time series.	A rate is a continuous variable and is appropriate for ordinary linear regression. Parameter estimates from ordinary linear regression are easily used to estimate attributable mortality. Age-specific rates adjust for changing age distribution.	We did not calculate population rates for influenza virology because we did not have age-specific virus detection counts.<br><br>Rates allow comparison between countries.<br><br>Populations were obtained from the Australian Bureau of Statistics.
For all-cause and combined respiratory and circulatory mortality, unable to fit a model that included the influenza B virology time series. Collinearity between seasonal influenza A and B was evident in some years (r>0.7 in 4 of 7 years).	Exclude influenza B from all-cause and combined respiratory mortality models.	The respiratory mortality model estimated negligible mortality for influenza B. Unlikely to be substantial for other outcomes.	Excluding influenza B did not appreciably alter the pandemic mortality estimate for 2009 and allowed a more plausible estimate for seasonal influenza A.

Table S2. Estimated number of influenza-attributable respiratory deaths, by influenza virus, model type and age, Australia, 2003 to 2009.

		Number (95% confidence interval)					
		Influenza A(H1N1)pdm09		Seasonal influenza A		Seasonal influenza B	
		Model		Model		Model	
Age group (years)	Year	Spline	Harmonic	Spline	Harmonic	Spline	Harmonic
<65	**2003**	n/a	n/a	137 (107, 166)	126 (90, 162)	0 (-1, 1)	0 (-1, 1)
	**2004**	n/a	n/a	133 (93, 173)	95 (47, 142)	-2 (-19, 16)	-1 (-21, 18)
	**2005**	n/a	n/a	67 (29, 105)	44 (-2, 90)	-3 (-26, 21)	-2 (-29, 25)
	**2006**	n/a	n/a	39 (-3, 80)	28 (-21, 78)	-3 (-31, 25)	-2 (-34, 30)
	**2007**	n/a	n/a	90 (55, 125)	90 (47, 132)	-2 (-23, 18)	-2 (-25, 22)
	**2008**	n/a	n/a	17 (-106, 140)	43 (-95, 180)	-12 (-120, 97)	-8 (-131, 114)
	**2009**	95 (63, 126)	97 (58, 137)	1 (-6, 8)	2 (-5, 10)	0 (-4, 4)	0 (-5, 4)
≥65	**2003**	n/a	n/a	655 (562, 748)	722 (597, 848)	0 (-3, 3)	-3 (-7, 0)
	**2004**	n/a	n/a	19 (-109, 147)	335 (168, 502)	2 (-54, 58)	-62 (-131, 7)
	**2005**	n/a	n/a	308 (186, 430)	245 (84, 407)	3 (-74, 79)	-85 (-179, 10)
	**2006**	n/a	n/a	53 (-82, 188)	137 (-37, 311)	3 (-89, 95)	-102 (-215, 12)
	**2007**	n/a	n/a	413 (299, 527)	411 (260, 563)	2 (-65, 70)	-75 (-158, 9)
	**2008**	n/a	n/a	223 (-184, 630)	662 (165, 1,158)	12 (-346, 370)	-396 (-838, 47)
	**2009**	152 (-82, 387)	-5 (-294, 284)	139 (-169, 447)	44 (-334, 422)	0 (-13, 14)	-15 (-31, 2)

Table S3. Estimated number of influenza-attributable respiratory and circulatory deaths, by influenza virus, model type and age, Australia, 2003 to 2009.

		Number (95% confidence interval)			
		Influenza A(H1N1)pdm09		Seasonal influenza A	
		Model		Model	
Age group (years)	Year	Spline	Harmonic	Spline	Harmonic
<65	**2003**	n/a	n/a	179 (103, 254)	230 (139, 321)
	**2004**	n/a	n/a	200 (104, 296)	119 (3, 234)
	**2005**	n/a	n/a	79 (-10, 169)	61 (-49, 170)
	**2006**	n/a	n/a	15 (-70, 100)	-32 (-137, 73)
	**2007**	n/a	n/a	179 (96, 262)	169 (68, 270)
	**2008**	n/a	n/a	-86 (-187, 15)	35 (-81, 151)
	**2009**	118 (36, 200)	30 (-68, 129)	-5 (-11, 1)	2 (-5, 9)
≥65	**2003**	n/a	n/a	1,369 (1,143, 1,596)	1,708 (1,389, 2,027)
	**2004**	n/a	n/a	447 (156, 738)	879 (468, 1,290)
	**2005**	n/a	n/a	1,216 (941, 1,490)	574 (182, 966)
	**2006**	n/a	n/a	263 (0, 525)	-109 (-489, 270)
	**2007**	n/a	n/a	978 (720, 1,236)	1,064 (697, 1,431)
	**2008**	n/a	n/a	921 (603, 1,238)	1,119 (694, 1,544)
	**2009**	231 (-340, 802)	-77 (-810, 657)	917 (208, 1,627)	-266 (-1,173, 641)

Note: Influenza B was excluded from the analysis for this outcome (see Table S1)

Table S4. Estimated number of influenza-attributable all-cause deaths, by influenza virus, model type and age, Australia, 2003 to 2009.

		Number (95% confidence interval)			
		Influenza A(H1N1)pdm09		Seasonal influenza A	
		Model		Model	
Age group (years)	Year	Spline	Harmonic	Spline	Harmonic
<65	**2003**	n/a	n/a	360 (201, 520)	395 (196, 595)
	**2004**	n/a	n/a	333 (130, 536)	145 (-109, 399)
	**2005**	n/a	n/a	-117 (-307, 73)	-78 (-319, 163)
	**2006**	n/a	n/a	-198 (-378, -18)	-88 (-319, 143)
	**2007**	n/a	n/a	312 (136, 487)	353 (131, 575)
	**2008**	n/a	n/a	124 (-91, 338)	207 (-48, 463)
	**2009**	29 (-144, 203)	26 (-191, 243)	7 (-5, 19)	12 (-3, 27)
≥65	**2003**	n/a	n/a	2,005 (1,664, 2,346)	2,240 (1,764, 2,716)
	**2004**	n/a	n/a	1,061 (622, 1,499)	1,084 (470, 1,698)
	**2005**	n/a	n/a	1,509 (1,096, 1,922)	191 (-394, 777)
	**2006**	n/a	n/a	251 (-145, 646)	-389 (-955, 178)
	**2007**	n/a	n/a	1,380 (992, 1,769)	1,377 (829, 1,925)
	**2008**	n/a	n/a	1,163 (685, 1,641)	2,019 (1,384, 2,653)
	**2009**	514 (-346, 1,374)	-280 (-1,375, 816)	921 (-148, 1,989)	-474 (-1,828, 881)

Note: Influenza B was excluded from the analysis for this outcome (see Table S1)

References for supplementary material

1. Louie JK, Acosta M, Winter K, Jean C, Gavali S, et al. (2009) Factors associated with death or hospitalization due to pandemic 2009 influenza A(H1N1) infection in California. JAMA 302: 1896-1902.
2. Donaldson LJ, Rutter PD, Ellis BM, Greaves FEC, Mytton O, et al. (2009) Mortality from pandemic A/H1N1 2009 influenza in England: public health surveillance study. Bmj 339.
3. Lee N, Choi KW, Chan PK, Hui DS, Lui GC, et al. (2010) Outcomes of adults hospitalised with severe influenza. Thorax 65: 510-515.
